# Methodological challenges in appraising evidence on diagnostic testing for WHO guidelines on hepatitis B and hepatitis C virus infection

**DOI:** 10.1186/s12879-017-2766-1

**Published:** 2017-11-01

**Authors:** Roger Chou, Philippa Easterbrook, Margaret Hellard

**Affiliations:** 1 Department of Medical Informatics and Clinical Epidemiology, Pacific Northwest Evidence-based Practice Center, Oregon Health & Science University, Portland, Oregon USA; 2 Global Hepatitis Programme, HIV Department, World Health Organization, Geneva, Switzerland; 3 Centre for Population Health, Burnet Institute, Melbourne, Australia

## Abstract

Linking persons with hepatitis B (HBV) and hepatitis C (HCV) infection with appropriate prevention and treatment requires that they first be diagnosed. The World Health Organization (WHO) has developed its first guidelines on testing for chronic HBV and HCV infection, using a framework based on methods from the Grading of Recommendations, Assessment, Development, and Evaluation (GRADE) Working Group for the formulation of recommendations, including determining the strength of recommendations and quality of evidence. Recommendations were formulated based on the overall quality of the evidence, in addition to other considerations, including the balance between benefits and harms, values and preferences, feasibility and resource implications. This article summarizes methodological challenges and additional considerations encountered in applying these procedures to diagnostic testing for viral hepatitis, and strategies to address these. Direct evidence on the effects of tests and test strategies on clinical outcomes was not available. Given the availability of effective treatments for HBV and HCV that are generally acceptable to patients, the Guidelines Development Group (GDG) considered diagnostic accuracy a reasonable surrogate for clinical outcomes. In order to increase the number of patients identified with chronic HBV and HCV infection who could benefit from treatments, the GDG determined that tests and testing strategies associated with slightly lower diagnostic accuracy could be recommended when associated with lower costs; increased testing access, uptake, and linkage to care; greater feasibility; or if preferred by patients.

## Background

Hepatitis B virus (HBV) and hepatitis C virus (HCV) are major worldwide causes of chronic liver disease and associated morbidity and mortality [1, 2]. Effective treatments are available for both chronic HBV infection as long-term suppressive therapy with tenofovir or entecavir and for HCV infection as curative short-term oral therapy with new direct-acting antiviral (DAA) regimens. However, most people infected with HBV or HCV are unaware of their status. As a result, opportunities are missed for earlier interventions and treatments to prevent or delay progression of liver disease and reduce risk of transmission and acquisition, and many present with advanced and irreversible liver disease.

Linking persons at risk of or with chronic HBV and HCV infection with appropriate prevention and treatment requires that they first be tested and know their status. The World Health Organization (WHO) recently developed its first guidelines on testing for HBV and HCV infection to inform national and regional scale up of testing. In the guidelines, the WHO addresses both *who* to test (i.e., which populations and where, and types of service delivery approaches) as well as *how* to test (i.e., which tests to use [3] and testing strategies and algorithms), and focuses on recommendations in low and middle income countries (LMIC). The guidelines are intended to complement other recently published WHO guidance on the prevention, care and treatment of chronic HCV and HBV infection [4, 5]. The target audience for these guidelines are national programme managers and other policy makers in ministries of health in LMICs who are responsible for the development of national hepatitis testing and treatment policy.

The guidelines were developed in accordance with the procedures developed by the WHO Guidelines Review Committee (GRC) [6]. The WHO procedures are based on methods from the Grading of Recommendations, Assessment, Development, and Evaluation (GRADE) Working Group to rate the strength of recommendations and quality of evidence [7]. Although GRADE has become a standard for developing evidence-based guidelines and has been widely adopted, applying GRADE for recommendations involving diagnostic testing [8] presented a number of methodological challenges. This article describes the methods used by the Guideline Development Group (GDG) to develop guidelines on HBV and HCV testing, key methodological issues in applying GRADE, and strategies used to address them that are relevant to the development of other guidance on the use of diagnostic tests.

## The GRADE (grading of recommendations, assessment, development, and evaluation) framework for formulating recommendations, grading the strength of recommendations and assessing quality of evidence

The WHO HBV and HCV testing guidelines were developed following the process described in the WHO *Handbook for Guideline Development*, 2012 [6]. The WHO process is based on the GRADE Working Group framework [9]. GRADE provides a process for evaluating and summarizing a body of evidence as well as formulating and grading recommendations based on that body of evidence. It is intended to make judgments regarding the quality of the evidence and the rationale behind recommendations clearer and more transparent by ensuring that reviewers and policy makers systematically consider and document all of the key issues relevant for assessing evidence and making recommendations. GRADE has been widely accepted and adopted by numerous systematic review and guideline development groups, and has been credited for providing a more rigorous and standardized system for guideline development [10].

## The WHO guidelines development process

In accordance with the approved GRC process, a GDG was formed with representation from various stakeholder groups, including researchers, clinicians and health programme managers, policy makers, advocacy groups, and members of organizations representing persons living with chronic hepatitis. Other factors used to inform the selection of GDG members included goals for diverse geographical representation and gender balance. Conflicts of interest were disclosed and reviewed for all GDG members and only persons who met the WHO conflict of interest policies were allowed to participate. In an initial scoping and planning process, a Steering Committee formulated the research questions most relevant to informing recommendations on HBV and HCV testing in LMICs, and prioritized the most important patient-centered outcomes [11]. The key questions were structured using the standard PICO (population, intervention, comparison, outcomes) format [11]. These questions were used as the basis for commissioning a series of systematic reviews and other studies to inform the guidelines development process. The systematic reviews used standard methods, including systematic literature searches, application of pre-defined criteria for inclusion and exclusion of the literature based on the PICOs, assessment of risk of bias, and synthesis of findings based on GRADE principles [9], with pooling of data with meta-analysis when appropriate. Overall, the formulation of recommendations was based on assessment of the quality of the evidence, the balance of benefits and harms, acceptability, resource use, and programmatic feasibility.

### Applying GRADE to evaluation of diagnostic testing for HBV and HCV infection

GRADE was originally developed to evaluate evidence and make recommendations about use of medical interventions, based on their effects on patient health outcomes (e.g., mortality, morbidity, function, quality of life) [9]. More recently, GRADE has also been adapted to evaluate and make recommendations regarding diagnostic tests [8]. Like medical interventions, the ultimate goal of diagnostic testing is to improve patient health outcomes [12, 13]. However, the quality and type of evidence available to evaluate diagnostic tests is often different from the ideal evidence (i.e., well-conducted randomized trials) used to evaluate medical interventions. Indeed, a major challenge in applying GRADE to diagnostic tests is that direct evidence on the effects of using or not using a test (or of using one test versus another) on clinical outcomes is often unavailable. Rather, most evidence on diagnostic tests addresses different measures of diagnostic performance, including both diagnostic (clinical) accuracy (e.g., sensitivity, specificity, and positive and negative predictive values and likelihood ratios) and analytical performance (e.g., linearity, precision, limit of quantification, limit of detection) [14–16] (Table 1). Diagnostic accuracy and analytical performance are related but separate concepts. Analytical performance refers to how reliably and accurately a test measures what it seeks to measure. Diagnostic accuracy refers to how well a test can distinguish between persons with the condition of interest from those without the condition. Tests with poor analytical performance are likely to have poor diagnostic accuracy, and assessments of diagnostic accuracy should generally follow demonstration of acceptable analytical performance. This article focuses on issues related to assessment of diagnostic accuracy, though analytical performance considerations are also discussed.

**Table 1:** Measures of diagnostic and analytical performance

Diagnostic/clinical accuracy	The ability of a diagnostic test/assay to correctly identify those with the infection or disease of interest and those without the infection or disease.
• Sensitivity	The ability of a test to correctly identify those with the infection or disease (i.e. true positives/true positives + false negatives).
• Specificity	The ability of a test/ assay to correctly identify the absence of the disease (i.e. true negatives/true negatives + false positives).
• Positive predictive value	The probability that when the test result is positive, the infection/disease is truly present (i.e. true positives/true positives + false positives). Predictive values are influenced by the prevalence of the disease in the population being tested.
• Negative predictive value	The probability that when the test result is negative, the infection/disease is truly absent (i.e. true negatives/true negatives + false negatives).
Analytical performance	The reliability and accuracy of a test/assay for measuring what it seeks to measure.
• Linearity	The degree to which the results of a test/assay are directly proportional to the amount of analyte.
• Limit of detection (LoD)	The lowest amount of an analyte that a test/assay can consistently distinguish from absence of the substance.
• Lower limit of quantification (LoQ)	The lowest amount of an analyte that a test/assay can consistently quantify.
• Precision	The degree to which repeated measures of an analyte by a test/assay under the same conditions provide the same results.

Adapted from World Health Organization Guidelines on Hepatitis B and C Testing, 2017, Glossary of Terms [3]

Diagnostic accuracy is an intermediate outcome because it only provides information about how well a test can determine whether a condition is present or not. In lieu of direct evidence on effects of diagnostic testing on clinical outcomes, guideline developers must use information on diagnostic accuracy to make inferences about potential effects on clinical outcomes, such as mortality, end-stage liver disease, and quality of life. Thus, even a body of evidence that provides strong evidence regarding diagnostic accuracy may provide only indirect and low quality evidence on effects on clinical outcomes, the basis for clinical recommendations. How well diagnostic accuracy can be used to predict effects on clinical outcomes depends on a number of factors, such as the availability of effective treatments, harms associated with the test and subsequent treatments, effects of test results on need for and use of additional testing (and any attendant harms), the impact of knowledge of test results on a patient’s well-being, access and linkage to care, testing coverage, and acceptability and uptake of treatments and subsequent testing [8]. The primary sources of evidence considered by the GDG included five systematic reviews on diagnostic accuracy of different tests [17–21] and one systematic review on interventions to enhance linkage to care [22]. The GDG also commissioned two surveys of hepatitis testing programmatic experience and feasibility [23–25], a survey on end-user values and preferences [26], and two narrative reviews on cost-effectiveness [24] to inform assessments of the feasibility, implementation, values/preferences, and costs/burdens associated with various testing strategies [27].

As anticipated, for evaluation of HBV and HCV testing, the WHO-commissioned systematic reviews found very limited or no evidence on effects on patient-important outcomes. Therefore, the primary basis for the GDG to develop recommendations was evidence on diagnostic accuracy. In general, testing for HBV and HCV is not associated with important direct harms and there are effective treatments for both HBV and HCV infection that are generally acceptable to patients. The GDG therefore determined diagnostic accuracy to be a reasonable surrogate for patient-important outcomes, given reasonable linkage and access to care.

#### Grading a body of evidence on diagnostic accuracy

A core principle in GRADE is the need to grade the quality of the evidence used to inform recommendations. For the purposes of developing guideline recommendations, GRADE defines quality as the confidence that the reported estimates of effect are adequate to support a specific recommendation [28], Following the GRADE framework, the quality of evidence was classified as high, moderate, low and very low, indicating the certainty in the estimates of effects (Table 2) [29].

**Table 2:** Key domains considered in determining the strength of recommendations^a^

Domain	Rationale
Quality of the evidence	Based on the presence of study limitations (risk of bias), inconsistency between studies, imprecision of estimates, indirectness, publication bias, and other factors. Higher quality evidence indicates greater certainty in the estimates and makes it more likely that a strong recommendation can be made.
Benefits and risks	Desirable effects (benefits) need to be weighed against undesirable effects (risks). The more that the benefits outweigh the risks, the more likely that a strong recommendation will be made.
Values and preferences (acceptability)	If the recommendation is likely to be widely accepted or highly valued, a strong recommendation will probably be made. If there are strong reasons that the recommended course of action is unlikely to be accepted, a conditional recommendation is more likely to be made.
Costs and financial implications (resource use)	Lower costs (monetary, infrastructure, equipment or human resources) or greater cost–effectiveness will more likely result in a strong recommendation.
Feasibility	If an intervention is achievable in a setting where the greatest impact is expected, a strong recommendation is more probable.

Adapted from World Health Organization Guidelines on Hepatitis B and C Testing, 2017, Table 3.2 [3]

^a^Recommendations were graded as “strong” (do in most circumstances) or “conditional” (do in many circumstances, but action may differ according to individual circumstances of the patient or setting)

Randomized controlled trials (RCTs) of interventions start as high-quality evidence, but can be downgraded for high risk of bias due to methodological limitations in the studies, inconsistency across study results, indirectness of evidence (due to use of indirect comparisons, intermediate outcomes, or evaluation of populations and interventions that differ from those of interest), imprecision and publication bias [5]. Observational studies of interventions start as low-quality evidence due to inherent methodological limitations, but may be upgraded if the magnitude of the treatment effect is very large, if evidence indicates a dose–response relationship, or if all important plausible biases would underestimate the observed effect*.*

In GRADE, observational studies of diagnostic accuracy (e.g., cohort and cross-sectional studies) can provide reliable evidence and are initially rated as high quality [8]. As with evidence on medical interventions, evidence on diagnostic accuracy can be downgraded due to risk of bias, inconsistency, indirectness, imprecision, and publication bias.

We summarise below how these criteria were applied to evaluation of studies on diagnostic accuracy of testing for HBV and HCV infection.

#### Risk of bias

The criteria used to assess risk of bias differ for studies on medical interventions and studies of diagnostic accuracy. For medical interventions, important factors include whether appropriate randomization and allocation concealment methods were used, similarity of groups at baseline, blinded administration of interventions and assessment of outcomes, loss to follow-up, and intention-to-treat analysis [30]. For studies on diagnostic accuracy, important factors include whether selection of patients may have introduced bias, the validity of the reference standard used, the time interval between the diagnostic test and the reference standard, whether pre-defined criteria for defining a positive test were utilized, and whether the results of the diagnostic test and reference standard were interpreted independently from one another [8]. For the HBV and HCV testing systematic reviews, the instrument used to assess risk of bias of studies on diagnostic accuracy was the Quality Assessment for Diagnostic Accuracy Studies-2 (QUADAS-2) tool [31]. The systematic reviews commissioned by WHO identified a number of methodological limitations in the diagnostic accuracy studies, such as failure to report whether patients were recruited consecutively, use of case-control designs, enrollment of predominantly hospital or facility-based patients or only healthy blood donors in some studies, convenience sampling or unclear patient selection methods, varying use of reference standards (e.g., use of enzyme immunoassay [EIA] and nucleic acid test [NAT] assays as the reference standard for studies of rapid diagnostic tests on detection of HCV RNA or HBV DNA [19, 20]), and failure to describe if the index and reference tests were performed in accordance with manufacturer recommendations and within 30 days on the same sample. Therefore, evidence on diagnostic accuracy typically warranted downgrading.

#### Inconsistency

Inconsistency refers to the extent to which studies evaluating the same comparison and outcome vary in their estimates. A challenge in evaluating bodies of evidence on diagnostic accuracy is that substantial inconsistency in estimates is commonplace [32]. For example, a review on HCV core antigen testing for diagnosis of HCV infection found that sensitivity estimates ranged from 44 to 100% [21]; inconsistency was also observed in other meta-analyses [17]. This inconsistency may be due to differences in populations, settings, the diagnostic tests, and threshold effects. Further, reliable methods to evaluate for statistical heterogeneity in pooled diagnostic accuracy estimates are lacking, making it difficult to determine the degree to which the evidence is consistent or inconsistent. The standard I^2^ statistic for evaluating statistical heterogeneity does not account for variations in estimates due to threshold effects or the correlation between sensitivity and specificity, which tends to lead to overestimation of the degree of heterogeneity [33]. Although the bivariate and hierarchical summary receiver operating characteristic (HSROC) random effects models for pooling data in meta-analyses provide an estimate of variance based on the tau-square parameter, this value is difficult to interpret because it is computed on an unfamiliar log odds scale [34]. Nonetheless, highly statistically significant tau-square values, as observed with pooled estimates in several of the diagnostic accuracy reviews, indicate that statistical heterogeneity is present.

#### Indirectness

Indirectness is defined in three main ways in GRADE: first, the degree to which patients and interventions in the studies differ from the specific ones of interest; second, when the outcomes differ from the patient centered health outcomes of interest; and third, when clinicians must choose between two or more interventions that have not been directly compared in head-to-head studies [35]. As noted earlier, evidence on diagnostic testing for viral hepatitis is characterized by indirectness in outcomes, since it focuses exclusively on diagnostic accuracy. Related to this, a potential source of confusion in the GRADE process is that the quality of evidence for a diagnostic test in a systematic review may be higher than the quality of evidence assigned to the recommendation. This is because for the systematic review, the quality of evidence is an indication of the confidence in the diagnostic accuracy estimate. This is not necessarily the same as the confidence in the evidence on patient health outcomes used to support a recommendation [8]. For example, even though the evidence on diagnostic accuracy of HCVcAg testing was assessed as consistent and precise, without serious limitations in quality, the evidence for the recommendation on HCVcAg testing was graded moderate quality, due to uncertain effects of slightly decreased diagnostic accuracy on clinical outcomes [20]. The presence of indirectness played a role in 14 of the 25 recommendations assessed as being based on low or very low quality evidence and 11 on low/moderate or moderate quality evidence, though other factors also contributed to the evidence grades.

#### Imprecision

Imprecision refers to the plausible range of values for outcome estimates, as typically indicated by 95% confidence intervals [36]. The importance of observed imprecision is based on the degree to which the recommendation would change if the upper versus the lower bound of the confidence interval represented the “true” value; if the recommendation would change, then the evidence is graded down for imprecision. This requires that a threshold be defined for what constitutes a clinically meaningful difference in diagnostic accuracy. For the viral hepatitis testing guidelines, imprecision could be defined a priori as clinically meaningful if there is a difference of 0.10 between the lower and upper bounds of the confidence interval for sensitivity or specificity. For example, a systematic review on HCV core antigen tests found that sensitivity for the Abbott ARCHITECT HCV Ag test was precise (93.4%, 95% CI 90.1 to 96.5%, difference between the lower and upper bounds of the confidence interval of 6.4%), but for the Ortho HCV Ag ELISA test it was imprecise (93.2%, 95% CI 81.6 to 97.7%, difference between the lower and upper bounds of the confidence interval of 16.1%) [21]. A systematic review of dried blood spot specimens for detection of HBV-DNA found that the estimate of sensitivity was precise (96%, 95% CI 90 to 98%) but the estimate for specificity was imprecise (99%, 95% CI 54 to 100%) [18]. It is worth noting that there is not a strong empiric basis for a threshold of 0.10 to define imprecision, and standards for clinically meaningful differences in diagnostic accuracy are not well-established.

#### Publication bias

Publication and related biases (e.g., reporting bias) refer to biases that occur because studies with certain results or particular outcomes are preferentially published, skewing the analysis [37]. For trials of medical interventions, registration in a clinical trials database allows reviewers to determine whether a study is unpublished and whether the pre-specified outcomes have been published, and to compare published results with the results entered in the registry [38]. However, similar, widely used registries are not available for diagnostic accuracy studies. Although graphical (e.g., funnel plots) and statistical methods are available for assessing for small sample effects that may indicate the presence of publication bias, the ability of the systematic reviews to evaluate for publication bias was limited by small numbers of studies for some analyses. Further, assessments for publication bias in meta-analyses need to account for threshold effects and the paired nature of sensitivity/specificity data, such as the trim and fill method based on the diagnostic odds ratio (defined as the positive likelihood ratio divided by the negative likelihood ratio) [39]. A systematic review of hepatitis C core antigen testing found no evidence of publication bias using this method for the Abbott ARCHITECT HCV Ag [21], but there were too few studies to formally assess for presence of publication bias for other test assays.

### Additional considerations for assessing diagnostic accuracy

#### Recommendations involving tests without a gold standard

In general, the systematic reviews focused on comparisons involving newer tests or test strategies against the “standard” test (e.g., rapid diagnostic tests versus enzyme immunoassay, HCV core antigen test versus nucleic acid testing, dried blood spot specimens versus standard venous specimens). Often, the diagnostic accuracy of the newer test or strategy was assessed as inferior to the standard test, but the quality of evidence was generally low, due to study limitations, inconsistency, imprecision, or other factors. As well, on many occasions there was no clear reference to assess the diagnostic accuracy of the “standard” test, which was often considered the “de facto” gold standard [3, 40, 41]. There was also a lack of data on the use of the newer diagnostic assays such as HCV core antigen for monitoring and verification of cure [3], and so no recommendation was made for its use in this context. In these situations, the GDG determined that strong recommendations for using the standard test were acceptable.

#### Clinical versus analytical diagnostic performance

The GDG focused on effects of diagnostic tests on diagnostic (clinical) accuracy. However, estimates of clinical accuracy are generally based on meeting assumptions regarding minimum analytical performance standards. This is a challenge for implementation given availability of a wide variety of commercially available HBV and HCV test assays, with variable testing and regulatory oversight. Depending on the specific assay used, actual test performance may differ markedly from what is observed in the studies included in the systematic reviews. Other factors that could impact test accuracy include differences across studies in the laboratory protocols used, including storage conditions (e.g., whether fresh samples were used) [17]. Therefore, for successful implementation of the viral hepatitis testing recommendations, the GDG recognized the importance of quality assurance in order that the tests used meet minimum performance, safety, and quality standards. The purpose of WHO’s prequalification program is to assist countries without the capacity to evaluate the quality and performance of diagnostic tests in insuring that tests meet technical and analytical performance standards [42].

Generally speaking, adequate analytical performance is a prerequisite for adequate diagnostic accuracy. In some cases, despite the absence of tests meeting analytical performance standard, diagnostic accuracy was not impacted. For example, for HBsAg RDTs, none of the studies in the systematic review met the limit of detection requirement (<0.130 IU/mL) from the European Union through its Common Technical Specifications; in fact, data from WHO prequalification assessment studies suggesting a limit of detection 50–100 higher (2–10 IU/mL) [3]. However, in this case, diagnostic accuracy was unlikely to be significantly reduced because the vast majority of chronic HBV infection is associated with blood HBsAg concentrations well over 10 IU/mL; this was reflected in the high accuracy of several RDTs versus EIA. There was also no data with which to compare the limit of detection between qualitative and quantitative HCV RNA or HBV DNA NAT assays or the minimum threshold for detection required [3]. However, with an increasing emphasis on diagnosis of viraemic infection and confirmation of cure with HCV, which can be done with qualitative tests, the need for a precise quantitative test has diminished as it is less relevant clinically.

### Other sources of evidence used to inform recommendations

#### Seroprevalence data

To inform recommendations on “who to test,” the WHO conducted background reviews on the global and regional seroprevalence of chronic HBV and HCV in LMICs, in the general population as well as specific populations [3]. The seroprevalence data was informative because based on a given estimate of diagnostic accuracy, testing in higher seroprevalence settings and populations will result in more efficient identification of persons identified. This in turn would be expected to result in more patients receiving antiviral treatments and other effective interventions. In the case of HBV and HCV testing, the GDG determined that the benefits of various testing strategies would increase at higher seroprevalences due to a higher yield (i.e., greater numbers of persons identified with viral hepatitis per number of tests performed), and therefore graded recommendations that focused on testing of the highest seroprevalence groups as strong.

#### Cost–effectiveness studies

To complement the data on seroprevalence, WHO commissioned narrative reviews on the cost–effectiveness of different HBV and HCV testing approaches, to further inform recommendations regarding “who” to test [24, 27]. It was not possible to undertake a formal systematic review and meta-analysis of cost–effectiveness studies because of the limited number of cost–effectiveness analyses and the heterogeneity of settings and populations studied (e.g. different risk groups in different countries), testing approaches used in different clinical and community settings; outcomes measured (e.g. infections detected, life-years [LYs] gained and quality-adjusted life years [QALYs] gained); and methods used to evaluate the cost–effectiveness of screening. Nonetheless, the studies generally indicated cost-effectiveness of testing, with increasing cost-effectiveness at higher seroprevalences, further supporting strong recommendations for testing in these populations.

#### Predictive modeling studies

Few studies directly compared effects of different testing strategies (i.e., one- versus two-test serological testing strategies) on diagnostic accuracy. Therefore, to inform recommendations on testing strategies, WHO commissioned predictive modelling studies that compared testing strategies to examine the accuracy of different testing strategies across a range of performance characteristics (i.e., sensitivity, specificity) of the assays (sensitivity and specificity), as well as a range of prevalence of the disease in the population (10%, 2%, 0.4%) representing high-, medium- and low-prevalence settings or populations, respectively [3, 43]. Different parameters were used for modeling chronic HBV and HCV infection. The models were used to estimate the number of infections identified relative to the number of tests performed and the number of false-positives and –negatives associated with alternative strategies. This type of evidence was not formally graded but was considered to be of low quality because it is very indirect and relies on a number of assumptions (e.g., degree of independence of each test utilized in a two-test strategy). Therefore, predictive modeling studies were not considered sufficient to upgrade conditional recommendations to strong.

#### Assessing end-user values, preferences, and acceptability

WHO also commissioned the development of a four-part online survey tool that examined current HBV and HCV testing practices and preferences for future HBV and HCV testing practices, including monitoring for HCV test of cure [23, 26]. The survey was undertaken in September 2015, and respondents included clinicians, patient organizations, civil society representatives, programme managers, policy-makers and pharmaceutical industry employees. The survey found that there were preferences for testing in high-risk settings, simple test strategies, and strategies that enable same-day testing.

#### Assessing viral hepatitis testing programmatic experience and feasibility

WHO also conducted interviews on experiences and reports of barriers/challenges to viral hepatitis testing in different settings and populations in LMICs, addressing testing programme information (who is tested and where, what assays/algorithms are used, counselling and training, funding and costs of testing); protocols for hepatitis care and treatment; perceived barriers/challenges and solutions; and provision of relevant epidemiological data. Finally, an innovation contest was undertaken to identify a range of different service delivery models of hepatitis testing practices in the field [44], in order to provide practical insights into implementation of testing, including services for key population groups. All of this information was used to inform the guidelines and the recommendation ratings.

#### Grading the strength of recommendations

In the GRADE system, the strength of each recommendation is rated separately from the quality of the evidence for that recommendation [9]. The strength of a recommendation indicates the extent to which the GDG was confident that the desirable effects of following a recommendation outweigh the potential undesirable effects. In WHO’s adaptation of GRADE, the strength of a recommendation is rated as “strong” or “conditional.” A strong recommendation is one for which the GDG was confident that the desirable effects of adhering to the recommendation clearly outweigh the undesirable effects. The implications of a strong recommendation are that they should be adopted in most circumstances for most patients. A conditional recommendation is one for which the GDG concluded that the desirable effects of adhering to the recommendation probably outweigh the undesirable effects, but the balance of benefits to harms was judged to be small or uncertain. The implications of a conditional recommendation are that, although most people or settings would adopt the recommendation, some would not, and other courses of action might be reasonable depending on the specific patient circumstances.

Factors supporting a strong recommendation include greater confidence in the anticipated effects (i.e., higher quality supporting evidence), large estimated benefits relative to harms, high acceptability of the recommendations to patients, lower resource use or greater cost-effectiveness, and high feasibility for implementation in the settings for which the recommendation is intended (Table 3) [45]. Reasons for making a conditional recommendation include a lack of high-quality evidence, small benefits relative to harms, and benefits that may not be worth the costs (including the costs of implementing the recommendation), or low acceptability to patients.

**Table 3:** GRADE categories of the quality of evidence

Level of evidence	Rationale
High	Further research is very unlikely to change our confidence in the estimate of effect.
Moderate	Further research is likely to have an important impact on our confidence in the effect.
Low	Further research is very likely to have an estimate of effect and is likely to change the estimate.
Very low	Any estimate of effect is very uncertain.

World Health Organization Guidelines on Hepatitis B and C Testing, 2017, Table 3.1 [3]

A challenge in developing the viral hepatitis testing guidelines was the lack of high quality evidence to support any recommendation. In general, GRADE suggests that strong recommendations should be avoided when the quality of evidence of low. However, for diagnostic tests, following such an approach would in the vast majority of cases lead to conditional recommendations, given that low quality (indirect) evidence is the norm. Although conditional recommendations for diagnostic testing are often appropriate, they also may be more difficult to implement, increase uncertainty, and may not accurately reflect judgments about likely benefits relative to harms. In the WHO viral hepatitis testing guidelines, nine recommendations were rated strong and 12 were rated conditional. As described above, some factors that informed strong recommendation ratings were high acceptability and feasibility, acceptable costs, supportive findings from predictive modeling studies, and whether the recommended test was the de facto gold standard.

#### Assessing trade-offs between benefits and harms

Given the availability of effective treatments for HBV and HCV infection, the GDG considered increased rates of testing and receipt of test results as a marker of additional patient health benefits because people who were aware of their infection would be more likely to receive treatment. In several cases, the GDG judged that the use of slightly less accurate diagnostic tests could be justified because the lower accuracy would be offset by increased uptake of testing and linkage to care. These recommendations were graded conditional because they involved trade-offs between lower diagnostic accuracy and increased access and linkage to care.

#### Future directions

Studies are needed to understand the effects of WHO testing recommendations on rates of testing, identification of persons with infection, linkage to care, and ultimately, clinical outcomes, in settings with different epidemiological patterns of viral hepatitis. In particular, research is needed to understand whether lower diagnostic accuracy for some conditionally recommended tests (e.g., RDTs [19], oral fluid tests [20], dried blood spot samples) is truly offset by greater uptake of testing and linkage to care. Studies are also needed to better understand harms of testing, including false-positives and –negatives, including the clinical importance of missed diagnoses among persons with occult or low-grade infection [19]. Interpretation of future studies on alternative or newer tests would be greatly facilitated by improved standardization of test assays and reference standards, and clear descriptions of testing indications, protocols, and characteristics of the populations evaluated. Research is also needed to determine how accuracy of diagnostic tests for HBV and HCV may vary in important subpopulations, such as HIV-infected persons [19] or in groups defined by age, sex, or risk factors for viral hepatitis. Studies are also needed to evaluate additional uses of newer tests, such as HCV core antigen as an alternative to viraemic assays for assessing response to treatment [21]. To ensure optimal implementation of the WHO recommendations, quality assurance efforts are needed to ensure that available tests meet performance, safety, and quality standards.

The GDG encourages efforts to further develop and standardize methods for developing recommendations on diagnostic testing and address methodological challenges such as how to determine when diagnostic accuracy is a suitable proxy for clinical outcomes, measure inconsistency across diagnostic accuracy studies, evaluate tests considered the de facto standard, incorporate modeling studies into recommendation decision frameworks, incorporate and weigh patient preferences and values in testing decisions, standardize methods for determining clinically important differences in diagnostic accuracy, and weigh potential trade-offs between diagnostic accuracy and improved access to testing and linkage to care.

## Conclusions

Identification of persons with chronic HBV and HCV infection is the critical first step for linking such persons to effective treatments and preventive strategies. To inform national programme managers and policy makers in LMICs who are responsible for the development of national hepatitis testing and treatment policy, WHO developed its first guidelines on HBV and HCV testing using the WHO GRC process based on methods developed by the GRADE Working Group. The GDG formulated its recommendations based on the overall quality of the evidence, the balance between benefits and harms, values and preferences, feasibility and resource implications. Modeling studies and surveys were used to supplement systematic reviews of diagnostic accuracy. Challenges in developing recommendations include the lack of direct evidence on effects of diagnostic tests and testing strategies on clinical outcomes, limitations in the evidence, and trade-offs between diagnostic accuracy and factors such as costs, increased testing access, and feasibility (Table 4). Given the availability of effective treatments for HBV and HCV that are generally acceptable to patients, the GDG considered diagnostic accuracy a reasonable surrogate for clinical outcomes. In order to increase the number of patients identified with chronic HBV and HCV infection who could benefit from treatments, the GDG determined that tests and testing strategies associated with slightly lower diagnostic accuracy could be conditionally recommended when associated with lower costs; increased testing access, uptake, and linkage to care; greater feasibility; or if preferred by patients. Despite the methodological challenges encountered in applying these procedures to diagnostic testing for viral hepatitis, the GDG found that GRADE principles could be applied to develop recommendations on who to test and how to test for chronic HBV and HCV infection that are based on the best available evidence and take into account important trade-offs, in order to improve the identification of affected patients.

**Table 4:** Key challenges in assessing evidence on diagnostic tests to develop recommendations on testing for HBV or HCV infection

• Need to rely on intermediate outcomes (diagnostic accuracy), requiring inferences regarding effects on clinical/patient outcomes
• Methodological limitations in diagnostic accuracy studies
• Inconsistency in diagnostic accuracy estimates, with lack of reliable methods for measuring statistical heterogeneity
• Imprecision in some diagnostic accuracy estimates
• Difficulty in determining accuracy of some standard assays due to the absence of an alternative reference standard
• No standardized/validated criteria for clinically important differences in diagnostic accuracy
• Wide variety of commercially available HBV and HCV test assays with variable testing and regulatory oversight
• How to incorporate/weigh findings from predictive modeling studies
• How to weigh trade-offs between lower diagnostic accuracy and lower costs, increasing testing access, uptake, and linkage to care; greater feasibility; and/or values and preferences
